# The Mystery at Silver Cliff

**Published:** 1876-07

**Authors:** Madge Nasheé


[*From Treasure Trove.*]

### *THE MYSTERY AT SILVER CLIFF.*

By Madge Nasheé.

I DO not think that I have ever seen a fairer creature than Daisy Leigh. Her hair was dead gold, which she wore rolled from her face, and coiled in a mass at the back of the prettiest little head in the world; great brown eyes, shaded by long, dark lashes; a nose slightly *retrousseé*, but none the less to be admired for that; and a mouth formed for kisses and laughter, but wearing the saddest, most touching expression imaginable.

And it was this very thing that first drew me toward her. She was so young, so pretty, and so inexpressibly sad, that my heart yearned to comfort her. She wore the deepest mourning, and we all knew that she had been terribly bereaved in the loss of her husband. For though not over twenty years of age, Daisy was a widow.

She came to the little hotel where I was spending the summer months one evening, accompanied by an elderly aunt, and had been with us some time without becoming acquainted with anyone, until one day I made a slight advancement, which she received so gratefully, that I was encouraged to proceed, and we soon became excellent friends; though it might seem strange that so young and attractive a person should take the slightest fancy to a crusty old maid like myself. I think it was because she saw my sympathy for her in my eyes, and my desire to win her confidence in my manner.

At any rate we became the best of friends in a very short time, and it was one evening in October, when we sat together in my cosy little parlor, with the moonlight faint and cold beaming through the lace curtains, and the firelight gleaming on her lovely, upturned face, that she told me her sad story, which I will relate in her own words:

"I was the only child of my parents, who were quite poor," said Daisy, slipping her hand in mine, "and who died before I reached the age of sixteen.

"I had received an excellent education through the generosity of my aunt, and after the death of my father, I became a governess in a family living in Quebec, Canada.

"It was there I met my husband." Her voice faltered; I pressed her hand, and in a few moments she continued. "It was very lonely, and Mark was a frequent visitor, and I soon learned to love him above all earthly things. It was he who procured for me the few pleasures I was allowed to enjoy; and one bright summer's morning—the very brightest and happiest of my life, I think—he told me how he loved me, and asked me to be his wife.

"I remember his dear face so well as he told me what was in his heart.

"'You have bewitched me, Daisy,' he said. 'From the first moment I saw you, I was a doomed man.'

"What could I do but put my hand in his, and say, 'Yes?'

"I knew that he was rich, and that I could bring him nothing but my love; but that was idle talk, he said—he wanted nothing but me. My employers thought he might have made a better match, I fancy; but they were very kind and generous to me.

"I learned Mark's exact position from them. His father had left him an ample property, which was burdened with the maintenance of a mother and sister, who resided a long way from Quebec, in a little settlement in Canada West. In addition to his property, Mark had an appointment under Government, a sort of surveyorship, the duties of which took him away to a great distance sometimes.

"'But I shall give it up as soon as we are married, my darling,' he said, 'and settle down with you at Silver Cliff.'

"That was the name of his place on the Ottawa River. My employer gave me many handsome and useful presents, and a good deal of advice. He told me that Mark Leigh was a good and upright man, he felt sure; but, for all that, he had heard reports about him which puzzled him very much, and which he thought I ought to hear.

"'What reports?' I asked.

"'Well, odd ones, to say the least of it,' he answered. 'Reports of dissipation and wild orgies that no decent young man would indulge in, and of other and darker deeds, which are only hinted at. I can not believe them, but it is strange that there should be even a whisper of a double life on the part of Mark Leigh.'

"I did not believe a word, and I said so. But oddly enough, the night before we were married, Mark himself spoke of the same thing.

"'Daisy,' he said, 'if ever you hear any evil of me, don't you believe it.'

"'What nonsense you are talking, dear,' I answered, 'of course I shall not.'

"'Not such nonsense as you fancy, my darling,' he said, with a sad look in his eyes.

"But I was too happy to wonder at his words, and the subject dropped between us forever. I came to understand afterward what it all meant, when it was too late—too late—and the knowledge could be of no use to me."

She shivered a little, and clung to me, as if the recollection of what she had endured was too overpowering.

"I will not tell you about my marriage, and our delightful journey to Mark's home. It was a pretty place, a perfect gem of a romantic nook, and to say that I was happy, but feebly expressed my feelings.

"We drove up to the gate in a little square wagon. I saw that there were farm buildings, looking trim and well-kept, and a couple of tidy servants standing behind their mistress when the door opened.

"My husband lifted me out of the wagon, and gave me to his mother, saying simply, 'This is my wife, mother;' and the old lady embraced me warmly, and so did her daughter. I returned their kisses, but somehow I felt afraid of them. Both their faces wore a set, hard look, and there was a kind of expectancy in their eyes, as though they were on the lookout for something. But they were very kind and tender to me, and seemed really glad to welcome me to their home. They took me to a sweet, pure chamber, all flowers and white draperies, and made me rest and refresh myself before I joined them down stairs. When I did so, it was in a pretty sitting-room, with pictures on the walls, and a soft, bright carpet on the floor, and we drank tea and chatted until in very deed I felt myself at home.

"Afterwards Mark took me the round of the pictures, and introduced me to his ancestors, near and remote, of whom he had a goodly collection. He was rather proud of his ancestors, one of whom had visited America in the train of Sir Francis Drake, and whose portrait graced the walls in all the dignity of ruff, stuffed sleeves, and other makings of a fine gentleman of his day.

"The portrait made an impression on me—it was so very like Mark. He laughed, and said the resemblance betrayed the purity of his descent, and the blue blood of the Leighs.

"'Sir Piers Leigh was the founder of our family, Daisy,' he said. 'There's a legend about him. I'll tell it you some time—a real ghost story, supported by the most unimpeachable testimony.'

"Another picture—I don't know why—made a curious impression on me. It was a beautifully-executed painting, as far as figures went, of a boy and girl—Mark and his sister when they were quite little children. The figures and faces were exquisite, but there was a want in the picture. The children were not looking at one another, but at the most clumsily-executed background I ever saw. It was like the picture on the case of a German clock—clumsy, ill-executed, and out of drawing.

"'Surely that was not all done by the same artist?' I said, looking at it closely.

"'No,' Mrs. Leigh said, gravely, for Mark did not answer at once. 'The picture was damaged, and we had recourse to the first man we came across, who called himself an artist, to set it to rights. He did his best, I suppose, but the result was not successful, I must say.'

"Her tone seemed to forbid further questioning, and I felt as though I had unwittingly touched upon an unpleasant topic. I felt uncomfortable altogether—I hardly knew why. My mother-in-law had welcomed me with loving smiles, and Laura, my new sister, had whispered to me how lovely I was, and how happy she was sure I had made Mark; but there was a chill somewhere. It was like being in a cosy chamber, radiant with light and warmth, and feeling an icy draught, the origin and direction of which is a mystery.

"I set it down to my being strange, and tired with my journey, and was not sorry when Mrs. Leigh proposed that I should go to my room. Laura went up stairs with me, and we sat talking a little while before she left me, saying that she would go down and send Mark up.

"Six months passed away, and all went smoothly on. Summer faded into autumn, and autumn chilled into winter, and my life promised to be as uneventful as it was happy. One night, just before Christmas, I opened the window of my room, and looked out. I did not feel sleepy, and such a glorious moonlight was flooding everything outside, making the snow-clad hills and valleys around look like a scene from fairyland. The broad river, which ran a very short distance from Silver Cliff, was frozen over, and in the white light of the moon looked like a sheet of molten silver. We were not so devoid of neighbors, but that I could see one or two houses here and there within reachable distance.

"There was a sleigh on the river, too—a solitary sleigh, with one person in it, coming along swiftly over the frozen stream, looking like a phantom thing. I watched it with curiosity. It did not look like any sleigh I had ever seen, and I was losing myself in conjectures as to what it could be, when I heard the voices of Mark and his mother underneath the window. They, too, had opened the window and gone out into the piazza, tempted by the beauty of the night. They were muffled in furs, and I heard Mark laughingly say something about their noses, and could tell that he was drawing something round his mother's face. Remembering all I had heard about Canadian winters, I hastily drew a woollen shawl about my head, and sat still. I never thought of drawing back. I was not listening particularly—rather lazily thinking that my husband's cigar smelt nice, as the light air wafted a faint smoke up to where I sat.

"'I see you have not told her, Mark?'

"It was Mrs. Leigh who spoke, and I knew instinctively she referred to me. Not told me what? I leaned forward to catch the answer.

"'No, mother.'

"'Was that well, my son?'

"'I think it was.'

"'I think not. She ought to know.'

"'Let it be for awhile,' he said gloomily. 'Why should I darken her young life with the story of the secret which has lined your face and whitened your hair—the miserable knowledge that sent my father to his grave a broken man, and makes our existence a constant dread of what may be? I would spare Daisy, if I could, mother. There may be no need to tell her at all.'

"'You know best, of course,' his mother answered; 'but we never know. Something dreadful may transpire here at our very door, and think of the shock—the humiliation.'

"'Don't, mother,' he said.

"And I could hear that she was weeping.

"'We will hope for the best. It is a long time since you saw or heard anything, isn't it?'

"'Many months; but that is nothing. It may be any time—to-night, for aught we know.'

"Laura gave a startled cry which interrupted her.

"'Look,' she said, 'there, on the river!'

"I had forgotten the sleigh in listening to what passed below, and I was startled to see it standing by the landing-place, and its occupant stepping out. He walked up to the house, and I saw that he was an Indian in full costume. I was terribly frightened. I had heard all sorts of stories of these people and their treachery, and this man might only be the forerunner of a whole bevy of them, come to murder and rob us. He walked silently up to the piazza where the others were sitting, his face quite visible in the moonlight, and I knew it. I was sure I had seen it before, though when or where I could not tell. He said a few words in a low tone to Mrs. Leigh, and her answer was a scream, that had a mournful, despairing ring in it. It was echoed by Laura, and I leaned right out of the window now to see, as well as to hear what was going on. Mrs. Leigh was stretching out her hands, as though to ward off a blow; Laura was clinging to Mark, who was regarding the intruder with an angry, but resolute face.

"'What new outrage is this?' I heard him say. 'What do you want?'

"'Money.'

"The word was spoken in perfectly good English, which astonished me, for I had heard that when the Indians spoke any language but their own, it was rarely anything else than Canadian French, and that in a broken sort of way.

"'You will get none here.'

"Mark spoke resolutely enough; but the Indian only laughed, and replied again in English—

"'I will.'

"'You will not.'

"'I will, if I call my braves, and burn the old place down about your ears. Such things have been.'

"I turned sick and cold as I thought of the atrocities I had read of, where settlers had been attacked by these 'noble savages,' their homes burned, themselves tortured and murdered for the amusement of their captors, and with difficulty I suppressed a scream. I did suppress it, and sat clinging to the window-sill, all ears; but I could not catch what further was said. To my surprise, they all went in-doors, taking the Indian with them, and I heard my husband's foot upon the stairs. He started at seeing me dressed, and at the window.

"'Not in bed, birdie?' he said; 'I thought you were asleep an hour ago!'

"'I couldn't; the moon was so bright until the last half hour,' I said; 'and now I'm so frightened. Oh, Mark!'

"'What is it, dear?'

"'That dreadful Indian. I heard him say——'

"'Oh, never mind what he said, dear,' Mark said, soothingly; 'I'm going to get rid of him. These gentry are very blustering in their demands. and this Mandomin, as he calls himself—it means the 'friend of man, Daisy, though he shows his friendship in a queer way—is about as blustering and objectionable as any Indian under the sun. We have to pay him a formidable amount of black-mail, or he might do us a mischief.'

"Mark spoke calmly enough, but there was an unquiet look on his face, and he was very pale. He was very much disturbed, I could see.

"'But are you sure he is an Indian?' I asked.

"'What, Mandomin? Yes, undoubtedly. Why?'

"'Because he spoke English.'

"'A rare accomplishment among them; but it occurs now and then. I fancy this particular specimen must have learned it in his childhood.'

"Still the same cheery voice and careless manner, with the troubled look and absent eyes, while he unlocked his desk, and took from it a roll of notes.

"'I am glad I was here when he came,' he said. 'My mother and sister are afraid of the Indians, though I tell them there is no real need; they are always to be bribed. I shall be up again in a few minutes, Daisy. Don't sit up any longer—it isn't safe for you in this cold.'

"He went down stairs, and in a very little while I saw the Indian leave the house, and embark again in his sleigh. Then Mark and his mother and sister came up-stairs, and I could hear the sobs and broken voices in which they bade him good-night. They were very much troubled by their nocturnal visitor, it was evident, but the emotion seemed more like grief than fright.

"Mark and I sat and talked for a little while. He evidently wanted to efface the impression of what occurred from my mind; and after awhile, with his arm around me and his loving eyes looking into mine, I forgot the past in the future, and chatted gayly with him about the plans we had laid for our life together.

"We were sitting on a broad, old-fashioned couch at the foot of the bed, he with his back to the door of the room, I so that I could see it. He was telling me of his resources, his position, and how at any time he could make a pleasant home for me anywhere if I found I did not like Silver Cliff well enough to stay there.

"'But I mean to like it, Mark,' I said, for I knew it was the wish of his heart that I should stay there. 'I like it already, and when I know your mother and sister better——'

"'You will love them as I do, dear. What is the matter—are you cold?' for all of a sudden I shivered as with an ague fit.

"'I think I am,' I said, for the room seemed to have grown curiously chill. 'Perhaps it was sitting at the open window.'

"'Ah, our Canadian nights are treacherous enough. I think I am a little cold myself. I don't think the fire's quite out; I'll make it up.'

"He would have risen to do so, but I stopped him with my hand. I could not have moved another inch or spoken a word to save my life. All my faculties seemed frozen into fright. Midway between the door and where we sat a man stood, clearly defined in the moonlight, for Mark's candle had gone out as he opened the door, and he had not relighted it. At first I thought of the Indian, but a second glance showed me this was not he. It was a man in the costume of some long-past period—doublet and hose, full ruff; he wore long gauntlets and a sword. It was as though the picture of Piers Leigh down stairs had walked out of its frame, and come upstairs to pay us a visit.

"'Daisy dear, what is the matter?' Mark said, alarmed at my wild looks.

"I could not speak—I could not move—and the appearance whatever it was, came slowly forward and touched Mark on the shoulder.

"'Ugh!' he said, with a shiver, 'there's a cold wind coming in. My dear child what is it?'

"For the man was gone now—we were quite alone, and I was clinging to him with incoherent gasps and helpless sobs I was quite unable to repress.

"'My poor child you have been frightened and worried,' he said; 'let me call my mother.'

"'No, no!' I cried. 'Take me away from here, Mark. This is a dreadful, haunted house.'

"'Haunted, Daisy?'

"I was in his arms, with his strong, circling clasp between me and danger, so I could tell him what I had seen. I saw every trace of color die out of his face as I spoke, leaving his very lips white.

"'It is cold,' he said, with a shiver. 'My poor pet, your head has been running on the Indian till you have fancied the other thing Things will appear all right in the morning.'

[TO BE CONTINUED.]

